# Healthcare providers' perceptions and experiences of kangaroo mother care for preterm infants in four neonatal intensive care units in China: a qualitative descriptive study

**DOI:** 10.3389/fpubh.2024.1419828

**Published:** 2024-07-08

**Authors:** Qian Cai, Yunxian Zhou, Mengxia Hong, Danqi Chen, Xinfen Xu

**Affiliations:** ^1^Department of Nursing, Zhejiang University School of Medicine, Hangzhou, Zhejiang, China; ^2^Women's Hospital School of Medicine Zhejiang University, Hangzhou, Zhejiang, China; ^3^School of Nursing, Zhejiang Chinese Medical University, Hangzhou, Zhejiang, China

**Keywords:** kangaroo care, neonatal intensive care unit, preterm, healthcare providers, qualitative study

## Abstract

**Background:**

Kangaroo mother care (KMC) is an evidence-based intervention that can effectively reduce morbidity and mortality in preterm infants, but it has yet to be widely implemented in health systems in China. Most qualitative studies on KMC for preterm infants focused on the experiences and influencing factors from the perspective of preterm infant parents, while neglecting the perspective of healthcare providers, who played a critical role in guiding KMC practice. Therefore, this study aimed to explore the perceptions and experiences of healthcare providers regarding their involvement in KMC implementation for preterm infants to promote the contextualized implementation of KMC.

**Methods:**

A descriptive qualitative approach was adopted. A purposive sampling was used to select healthcare providers involved in KMC implementation in the neonatal intensive care units (NICUs) as participants from four tertiary hospitals across four cities in Zhejiang Province, China. Face-to-face semi-structured interviews were conducted to collect information. Thematic analysis was employed to analyze the data.

**Results:**

Seventeen healthcare professionals were recruited, including thirteen nurses and four doctors in the NICUs. Four themes and twelve subthemes emerged: different cognitions based on different perspectives (acknowledged effects and benefits, not profitable economically), ambivalent emotions regarding KMC implementation (gaining understanding, gratitude and trust from parents, not used to working under parental presence, and concerning nursing safety issues), barriers to KMC implementation (lack of unified norms and standards, lack of systematic training and communication platform, insufficient human resources, and inadequate parental compliance) and suggestions for KMC implementation (improving equipment and environment, strengthening collaboration between nurses and doctors, and support from hospital managers).

**Conclusions:**

Despite acknowledging the clinical benefits of KMC, the lack of economic incentives, concerns about potential risks, and various barriers hindered healthcare providers' intrinsic motivation to implement KMC in NICUs in China. To facilitate the effective implementation of KMC, hospital managers should provide bonuses and training programs for healthcare providers, while giving them recognition and encouragement to enhance their motivation to implement KMC.

## 1 Introduction

According to the World Health Organization (WHO), an estimated 13.4 million preterm babies were born globally in 2020, accounting for 9.9% of total newborns (1, 2). In China, the overall incidence of preterm birth increased from 5.9% in 2012 to 6.4% in 2018 (3), and the rate of low birth weight also rose from 2.34% in 2010 to 3.70% in 2021 (4). Notably, in 2021, the low birth weight rate in Zhejiang Province was 4.48% (4), higher than the national average. It was indicated that the neonatal mortality rate in China had declined from 22.8% in 2000 to 3.1% in 2021 (4). Furthermore, the neonatal mortality rate in Zhejiang Province had dropped from 2.75% in 2010 to 1.16% in 2020 (5). From 2009 to 2018, the proportion of preterm births among newborn deaths in China increased from around 42.6–49.8% (6). Immaturity, asphyxia, and congenital abnormalities accounted for approximately 80% of preterm infant deaths (6). Preterm birth complications remained a leading cause of neonatal mortality in China (7), indicating that clinical care for preterm infants was still insufficient (8). Many survivors faced lifelong disabilities, including learning disabilities, sensory impairments, and motor disorders (9–11). In 2022, WHO updated its recommendations for preterm care, suggesting that Kangaroo mother care (KMC) should be initiated immediately after birth, which could significantly improve preterm infants' survival and health outcomes (12).

KMC refers to a clinical care approach in which the mother (or the father) of a preterm infant holds the naked baby against her (or his) bare chest in the same way as kangaroo parenting (13). This method allows for early, continuous, and prolonged skin-to-skin contact between the preterm infant and the parent, while also promoting exclusive breastfeeding and measures such as early discharge and post-discharge follow-up (14, 15). Compared to traditional care, KMC has numerous short-term and long-term benefits for newborns, such as stabilizing physiological status, promoting neurobehavioral development, enhancing immune function, increasing exclusive breastfeeding rates, and fostering attachment between the newborn and caregivers (16–19). Despite extensive research reporting on the benefits and effectiveness of KMC, this intervention has not been fully integrated into healthcare systems globally (20, 21).

China is confronted with one of the highest rates of preterm birth, a trend that continues to escalate annually (1, 3). In 2014, the National Health Commission of China's Department of Maternal and Child Health initiated the Premature Birth and Preterm Infants Intervention program, enlisting ten hospitals to pioneer the implementation of KMC (22). In 2017, the commission issued the “Guidelines for Health Care Services for Premature Infants,” which catalyzed the broader adoption of KMC within the country (23). Building on this momentum, the “Action Plan for Healthy Children (2021–2025)” (24), published by the National Health Commission, underscored the importance of promoting kangaroo care as a means to enhance the quality of life for premature infants. Despite these concerted efforts and significant strides, the practice of KMC has yet to achieve widespread adoption in China. Specifically, while the neonatal intensive care units (NICUs) of tertiary referral hospitals related to maternal and child health in China all provide level III of neonatal care (25), a survey revealed that the current adoption rate of KMC in these NICUs was mere 21.2% (22). This figure indicated that although some hospitals in China had piloted KMC in their NICUs to varying degrees, it did not become routine practice (26), and the scale-up of KMC implementation remained sub-optimal. Factors influencing KMC implementation primarily included environmental factors, parent/family factors, access factors, cultural factors, and professional factors (27). Among these factors, for healthcare providers, support from leadership, training programs on KMC, and adequate staffing were key to KMC implementation. Feucht et al. pointed out that healthcare providers played an important role in KMC (28). To some extent, they were the supporters and supervisors in KMC practice, requiring them to have the knowledge, experience, and willingness to implement KMC (29).

Previous qualitative studies on KMC for preterm infants primarily focused on parents' experiences of participation and the influencing factors to its implementation from their perspective (30–32). However, participants in these studies indicated that the information provided by healthcare professionals significantly influenced their experiences of engaging in KMC with their preterm infants. Therefore, it was crucial for healthcare providers to recognize the value of KMC and to establish strong relationships with parents to effectively explain the necessity and effectiveness of KMC, which would potentially enhance parents' adherence to KMC implementation (33). A study on the barriers and facilitators to KMC revealed that KMC implementation was not running optimally due to inconsistent local leadership, heavy workload, and the knowledge and attitudes of health workers (34). Feng et al. directly pointed out the importance of research conducted from the perspective of healthcare professionals and recommended exploring their views and suggestions on KMC to facilitate its implementation (35). It was evident that gaining insights into healthcare providers' cognitive and practical experiences of KMC, using a qualitative approach, was crucial in advancing the implementation of KMC.

However, research exploring the perceptions and experiences of healthcare providers regarding KMC was relatively limited. A qualitative study by Pratomo et al. indicated that due to the lack of systematic and formal KMC training, healthcare providers had misconceptions about certain aspects of KMC, which could impact the successful implementation of KMC (36). Some nursing staff mentioned in a study that despite improving their knowledge about KMC, they were still confused about its safety and appropriate application (37). On the other hand, Rahmatika et al. indicated that healthcare providers held positive perceptions of KMC and believed that this positive attitude was crucial for providing KMC education to facilitate its implementation (38). Only one study in China conducted a preliminary exploration of NICU nurses' participation in KMC, highlighting the shortage of nursing resources as a barrier to KMC implementation and emphasizing the importance of standardized language in communication, operational homogenization, and standardized training (39). However, the study was conducted in only one hospital and was limited to the real feelings and experiences of nurses without exploring their views and cognition on KMC implementation. Overall, there was a notable gap in research concerning the perceptions and experiences of NICU healthcare providers regarding implementing KMC.

This study aimed to conduct interviews with healthcare providers who implemented KMC for preterm infants in the NICUs of four tertiary hospitals across four cities in Zhejiang Province, China, in order to deeply explore their feelings and experiences in guiding parents of preterm infants to practice KMC, as well as their perceptions of KMC and the challenges they faced. This helped us comprehend the current situation of KMC practice in China from the healthcare perspective, enabling us to better promote the customized implementation of KMC in the future and providing a reference for KMC application in NICUs with similar restricted visitation policies in other developing countries.

## 2 Materials and methods

### 2.1 Study design

A descriptive qualitative design was adopted. This approach is appropriate for research questions that aim to provide the most direct and essential answers to the concerns of practitioners or policymakers, which enable new insights to emerge that provide a richer understanding of the phenomenon (40). This study is reported in accordance with the consolidated criteria for reporting qualitative research (COREQ) guideline (41).

### 2.2 Setting and participants

This study was carried out in the NICUs of four tertiary hospitals in Hangzhou, Shaoxing, Jiaxing, and Huzhou in Zhejiang Province, China, all of which had previously implemented KMC. These hospitals were chosen for the study based on their high utilization of KMC and accessibility. Initially, the first author directly contacted the head nurses of the NICUs in each hospital, explaining the research objectives and obtaining their consent to participate in the study. In each ward, the head nurse was assigned as the study coordinator for that site and assisted in recruiting a mix of doctors and nurses who had experience with KMC for interview. There had been no prior relationships between the researcher and these participants. However, we faced several challenges during recruitment, primarily due to the time constraints of the healthcare providers and their initial hesitancy to participate in research activities. To overcome these challenges, we established open communication with the healthcare providers in the NICUs, providing clear information about the study's aims and ensuring confidentiality. Additionally, we offered flexible scheduling for interviews to accommodate the participants' availability.

This study employed a purposive sampling method to select participants. The method effectively targets individuals with similar experiences based on predetermined criteria within a specific environment, thereby gathering comprehensive data for the study (42). Moreover, the selection process aimed to ensure the representation and diversity of participants by considering various factors like age and years of professional experience. All participants had to meet the following inclusion criteria: nurses and doctors both needed to be licensed; NICU working experience ≥ 5 years; having experience in KMC; proficiency in expression abilities. Exclusion criteria for healthcare providers included individuals in rotating, internship, trainee, or residency positions.

### 2.3 Data collection

Qualitative data were collected using face-to-face, semi-structured individual interviews conducted in four of the NICU wards from January to April 2023. Demographic details of participants were collected prior to the interview using self-designed questionnaires. The first author designed the initial interview outline, and after two pilot interviews and internal discussions, the semi-structured interview guide was revised and adjusted (43), mainly to ensure the questions could be easily understood by the interviewees (Supplementary Tables S1, S2).

Interviews were conducted in private and calm rooms within the healthcare facilities so that the participants would be more comfortable expressing themselves. At the outset of each interview, the primary researcher introduced herself and asked introductory questions to establish rapport and put participants at ease. Each interview lasted ~40–70 min, and all the interviews were conducted in Chinese and audio-recorded with the participant's consent. There were no repeat interviews. Field notes were taken and used to capture interview details, including non-verbal communication.

### 2.4 Data analysis

Data analysis occurred concurrently with data collection. All audio recordings were transcribed verbatim for the initial transcripts. These initial transcripts were manually verified against the recording for fidelity by the first two authors, and then managed using NVivo 12 software (QSR International, Melbourne, Australia). Participants were assigned codes to maintain confidentiality.

Data analysis was conducted using thematic analysis guided by Braun and Clarke's approach (44). The analysis process comprised the following six steps: initial codes were developed by two researchers after independently reading the first four transcribed interviews multiple times; through in-depth analysis of the remaining 13 interview transcripts, initial sub-themes and themes were developed; the researchers continued to enhance the sub-themes and themes through iterative refinement; the research team collaborated to refine the representation of sub-themes and themes, ensuring alignment with the research question and establishing the final thematic framework; all sub-themes and themes were subjected to review by the research team in order to establish consensus on clear definitions and names; the data analysis findings were presented in a narrative format by the first author, and subsequently confirmed by the research team.

The data analysis was conducted in Chinese, and selected thematic codes were translated into English for presentation in the Section 3. In order to avoid any misinterpretation or inaccurate translation, a rigorous process involving forward translation, back-translation and reconciliation of discrepancies was employed. The initial forward translation and the back-translation were conducted by the first author interviewer (QC), a female PhD candidate fluent in Chinese and English. Any differences that emerged during the back-translation were carefully reviewed and reconciled by the first co-author (YZ), who obtained her PhD degree in Australia and currently serves as a doctoral supervisor at a university in China. Quotations connected with each of these themes were selected and utilized to highlight the various fashions in which these participants described themes. Saturation was achieved when new information from interviews became limited, indicating the identification of all relevant themes and concepts (45). Specifically, after the 15th interview, there were no new themes generated from the interviews in our study. To ensure that we achieved data saturation, we proceeded with two additional interviews to ensure and confirm that no new themes were emerging. The pilot interview data were not included in the analysis.

### 2.5 Rigor

A qualitative study is commonly evaluated by trustworthiness, including credibility, transferability, dependability, and confirmability (46). In this study, credibility was established through peer debriefing, in which the researchers consulted with one another to address any ambiguities or disagreements on methodological issues or data analysis. Transferability was attained by the transparent method, which might allow auditing and replicating to occur in other settings. Dependability was obtained using verbatim transcriptions, detailed field notes, and a record of analytical decisions, as it provided an audit trail that could be reviewed and verified. Confirmability was obtained using interview quotations to illustrate the informants' voices in the results section.

### 2.6 Ethical considerations

Ethical approval was obtained from the Ethics Committee of the Women's Hospital, School of Medicine, Zhejiang University (Approval No. IRB-20220219-R) before the start of the study. Written informed consent was provided by each participant before the interviews. All participants could withdraw from the study at any stage without disclosing the reason. The audio recordings and transcripts were tagged with pseudonyms so that confidentiality and anonymity were assured and data were safely archived in files protected by passwords.

## 3 Results

A total of 17 healthcare providers in the NICUs were interviewed in this study, including four doctors and 13 nurses, with four nurses being head nurses. Among them, two doctors were male, while the rest of the participants were female. The participants ranged from 33 to 52 years old, with an average age of 41.2 years, and they had an average work experience of 19.7 years (11–33 years), with an average of 14.5 years of experience working in the NICUs (9–24 years). All participants held a bachelor's degree or higher and possessed intermediate or higher professional titles. The detailed characteristics of the participants are presented in Table 1. The analysis yielded four main themes and twelve subthemes, as outlined in Table 2.

**Table 1:** Characteristics of the healthcare providers interviewed (*N* = 17).

**No**.	**Gender**	**Age (years)**	**Working experience (years)**	**Working experience in NICU (years)**	**Education level**	**Professional title**	**Position**
H1	Female	46	28	12	Bachelor	Senior title	Head nurse
H2	Female	35	12	12	Master	Medium-grade title	Staff nurse
H3	Female	40	19	19	Bachelor	Medium-grade title	Nurse team leader
H4	Female	43	25	24	Bachelor	Medium-grade title	Staff nurse
H5	Male	35	12	12	Bachelor	Medium-grade title	Medical team leader
H6	Female	46	27	10	Bachelor	Senior title	Head nurse
H7	Female	40	17	15	Master	Medium-grade title	Staff nurse
H8	Female	38	12	11	Master	Medium-grade title	Staff doctor
H9	Female	35	13	12	Bachelor	Medium-grade title	Staff nurse
H10	Female	44	26	21	Bachelor	Medium-grade title	Nurse team leader
H11	Female	52	33	15	Bachelor	Senior title	Head nurse
H12	Female	47	26	12	Bachelor	Senior title	Staff doctor
H13	Male	40	11	11	PhD	Senior title	Medical team leader
H14	Female	41	21	21	Bachelor	Medium-grade title	Nurse team leader
H15	Female	40	19	12	Bachelor	Medium-grade title	Head nurse
H16	Female	33	13	9	Bachelor	Medium-grade title	Staff nurse
H17	Female	45	20	18	Master	Medium-grade title	Nurse team leader

NICU, neonatal intensive care unit; PhD, doctor of philosophy.

**Table 2:** Themes and subthemes.

**Themes**	**Subthemes**
Different cognitions based on different perspectives	Acknowledged effects and benefits
	Not profitable economically
Ambivalent emotions regarding KMC implementation	Gaining understanding, gratitude and trust from parents
	Not used to working under parental presence
	Concerning nursing safety issues
Barriers to KMC implementation	Lack of unified norms and standards
	Lack of systematic training and communication platform
	Insufficient human resources
	Inadequate parental compliance
Suggestions for KMC implementation	Improving equipment and environment
	Strengthening collaboration between nurses and doctors
	Support from hospital managers

KMC, kangaroo mother care.

### 3.1 Theme 1: different cognitions based on different perspectives

Differences in cognition on KMC were observed among healthcare providers. The majority of the participants believed that KMC could benefit preterm infants and their parents, including improving and stabilizing the infants' conditions, shortening their hospital stays, and alleviating family members' emotions. However, most healthcare providers also believed that, from the perspective of hospital revenue, the relatively low fees for KMC were essentially non-profitable compared to the time and effort they dedicated.

#### 3.1.1 Acknowledged effects and benefits

The majority of healthcare providers [13 participants (76%)] endorsed the significant benefits of KMC for preterm infants. They pointed out that KMC could enhance and stabilize the physiological wellbeing of preterm infants, including breathing, heart rate, and body temperature. Moreover, KMC was observed to facilitate weight gain in preterm infants and better promote their health and development by providing a sense of security and close contact with their parents.

Many of the preterm infants with underdeveloped lung development experienced desaturation and respiratory distress. However, when they were placed on their mother's chest, they did not need oxygen. (H6)

Some healthcare providers [five participants (29%)] also mentioned a notable association between the KMC and reduced hospitalization time for preterm infants. The adoption of KMC promoted the turnover of NICU beds to free up valuable bed resources to care for other preterm infants in need, which was particularly crucial in NICUs experiencing bed shortages or in hospitals undergoing diagnosis-related group system (DRGs) assessment. Moreover, by shortening the duration of hospitalization, KMC decreased the risk of nosocomial infection among preterm infants.

Especially for now, there is also an assessment based on DRGs. If the length of hospital stay is too long, the costs will be higher, resulting in a loss… KMC can shorten the hospital stay of preterm infants, reducing bed occupancy and thereby increasing bed utilization rates, indirectly promoting the revenue of the NICU… There is a heightened risk of infection for a preterm infant who is unable to be discharged for a prolonged period. (H3)

Moreover, some healthcare providers [three participants (18%)] highlighted that KMC had additional effects on parents of preterm infants and hospitals. During KMC, parents actively participated in caring for their preterm infants and observed their infants' condition and changes firsthand, which could alleviate their negative emotions, foster communication between parents and healthcare providers, and potentially enhance the hospital's reputation positively.

Enabling parents to witness their preterm infants visually helps alleviate their anxiety and pressure. It can also minimize complaints, promote the hospital's reputation, and reduce potential disputes and conflicts. (H13)

#### 3.1.2 Not profitable economically

Despite the benefits KMC brought to families of preterm infants and hospitals, the economic revenue it could gain was a consideration. As an emerging nursing care practice, the fees for KMC needed regulation by the Price Bureau, that is the fees should be set according to specific standards rather than arbitrarily based on costs incurred. Some healthcare providers [six participants (35%)] believed that the fees for KMC were relatively low, and the current charge standards did not reflect the actual costs of the services and resources provided by healthcare workers.

It's really cheap, not covering the costs because our KMC is priced at just 120 RMB per session, but as far as I know, the effort our nurses put in far exceeds the value of this amount. (H17)

In contrast to low fees, department costs for KMC implementation were high. Many healthcare providers [nine participants (53%)] mentioned that implementing KMC was basically a financial loss compared to the substantial time and effort dedicated by medical staff. Despite KMC being predominantly carried out by parents of preterm infants, medical staff's guidance, collaboration, and support were deemed crucial, thereby further increasing their workload. For NICUs, the extra costs incurred by the devotion of medical staff may not have been fully compensated through KMC charges.

Basically, the nurse spends almost two hours for each session. The income generated is very little. In terms of the labor costs, doing KMC is actually a loss. Spending the entire morning there only earns a few tens of yuan for the department. That's why many hospitals are reluctant to do it. (H1)

### 3.2 Theme 2: ambivalent emotions regarding KMC implementation

The emotional experience of healthcare providers involved in KMC was ambivalent. They mentioned that receiving positive feedback from parents made them feel their efforts were acknowledged and appreciated, which brought them a sense of accomplishment and fulfillment. However, they still felt uncomfortable and uneasy about the working pattern with parents present, and they also worried about potential risks or adverse effects that might arise from KMC. This sense of contradiction permeated their involvement in implementing KMC.

#### 3.2.1 Gaining understanding, gratitude and trust from parents

During their participation in KMC, parents could direct skin-to-skin contact with their preterm infants and receive guidance on caring for infants from healthcare professionals, such as feeding. Some healthcare providers [five participants (29%)] expressed that they could sense the increasing satisfaction from parents and the gradual improvement in cooperation with each other. Gradually, a strong emotional connection was established, further enhancing the relationship between healthcare providers and parents. They mentioned that while supporting parents participating in KMC, they received positive emotional feedback from parents occasionally. The most important aspect of these gains was the understanding, gratitude, and trust, which made them feel their work was acknowledged and recognized.

Tears welled in her eyes at the moment of skin-to-skin contact, feeling truly touched as she held her infant close. She was very grateful to us… If we assist her effectively, she will be extremely thankful. (H10)

When parents witness firsthand and perceive the dedication you put into ensuring the well-being of their babies during this process, their sensation is profoundly intuitive, and then they will have a deeper level of trust. Many relationships are built in this way. (H11)

#### 3.2.2 Not used to working under parental presence

With the restricted visitation system in NICUs, healthcare professionals had adapted to working without parents present. Implementing KMC meant that their procedures and communications were exposed to the parents. Some healthcare providers [seven participants (41%)] expressed feeling anxious and stressed due to the perceived scrutiny and monitoring by parents. They were concerned that parents might question their professional competencies or the quality of care, causing them to be highly vigilant in their words and actions to avoid criticism or reproach.

Our nurses were used to working without the presence of parents. Having parents involved in KMC definitely adds pressure on us because they would observe all our behaviors. We all know that our operations are not always perfect, which is impossible, but parents may not think it that way. (H2)

Moreover, in the era of highly advanced modern information technology and networking, parents present at the scene might take photos, record audio or videos to document specific medical procedures or even detailed conversations among healthcare providers. Some healthcare providers [five participants (29%)] mentioned that once these recordings were disseminated and magnified, they could pose hidden risks of medical disputes. Even inadvertent errors or negligence could be scrutinized and distorted later, potentially damaging their reputation and professional careers.

Parents may misunderstand if healthcare workers deviate slightly from standardized procedures in some operations or even the things mentioned in our casual conversations. This information might be exposed online or shared in other ways, so we need to be very cautious. It makes us feel nervous internally. (H4)

#### 3.2.3 Concerning nursing safety issues

Parents' involvement in KMC entailed their entry into the NICU. Many healthcare providers [eight participants (47%)] were concerned that some parents might be in the latent period of infection or carry pathogens. Additionally, parents typically did not undergo strict disinfection procedures before entering the NICU. Some items that had not been disinfected, such as phones, keys or wallets, could also have carried pathogens, thereby increasing the risk of infection for preterm infants.

There are still some risks in the entire process, and we fear the possibility of hospital-acquired infections. This is actually our biggest concern. (H3)

Some healthcare providers [four participants (24%)] expressed that inadequate observation and delayed responses in the process of KMC could potentially result in adverse events. This was particularly concerning when there was a shortage of nursing staff and lapses in care. For instance, preterm infants might experience a drop in oxygen saturation during feeding. If nurses do not notice this promptly, it could lead to complications. Similarly, during skin-to-skin contact, preterm infants could accidentally suffocate due to their immature respiratory systems.

Especially those infants who require oxygen therapy are inherently unstable. If their condition suddenly changes during this process, even if we come over to deal with it immediately, it may still cause harm to the infant. (H11)

### 3.3 Theme 3: barriers to KMC implementation

Healthcare providers indicated that they faced some practical obstacles in implementing KMC. The lack of standardized protocols might affect its effectiveness and safety. The absence of systematic training for them could impact the quality of KMC implementation. Additionally, insufficient manpower and parental compliance might affect its successful implementation.

#### 3.3.1 Lack of unified norms and standards

Many healthcare providers [10 participants (59%)] indicated that hospitals in China had not established unified, standardized KMC implementation protocols, leading to variations in operational norms or execution standards among hospitals. Due to the lack of standardization, the implementation process of KMC could easily become a more subjective care-giving behavior, making it challenging to ensure the quality and safety of KMC and bringing about confusion and uncertainty for healthcare providers.

Currently, KMC may have its own standards in each hospital, but it has not formed a relatively unified operational process like many other procedures, so there is no clear execution standard. (H9)

#### 3.3.2 Lack of systematic training and communication platform

Some healthcare providers [six participants (35%)] mentioned that hospitals generally lack systematic training on KMC. If KMC was not regularly implemented, some details might be overlooked. However, some NICU departments believed they had been conducting KMC for a long time and were already proficient without encountering adverse events during implementation. Consequently, they underestimated the importance of regular, systematic KMC training.

There is no specialized training available for KMC. In reality, it is needed. Everyone is very busy with their work, and if something is posted in the DingTalk work group, they may not pay much attention to it. (H12)

Some healthcare providers [five participants (29%)] also mentioned the absence of synchronous experience-sharing and learning channels. Currently, there is no specialized platform or website for KMC communication, making it difficult for them to exchange experiences and engage in online learning activities. This hindered the improvement of KMC implementation quality and the timely adoption and application of the latest advancements and progress.

Training and communication regarding KMC for preterm infants are still relatively scarce. We are not quite sure about the current situation and how well-established hospitals are approaching this. (H16)

#### 3.3.3 Insufficient human resources

The majority of healthcare providers [12 participants (71%)] indicated that participating in KMC directly increased their workload, which emerged as a primary challenge. Consequently, effective implementation became difficult when the departments were busy or under circumstances of limited nursing staff. The low nurse-to-patient ratio imposed a heavy workload on healthcare providers, constraining their ability to allocate the necessary time and energy for the high-quality implementation of KMC. This scarcity of personnel not only hindered the effective execution of KMC but also potentially influenced healthcare providers' attitudes toward and willingness to implement KMC.

The nurse-to-patient ratio is insufficient. One nurse has to care for 10 infants or even more, and we really don't have enough time to spare. We can't even complete our daily tasks, so how can we do it? That's why we have to assign additional nurses specifically for KMC. (H15)

Furthermore, some healthcare providers [five participants (29%)] highlighted that implementing KMC required a high level of nursing competence. It frequently needed the support of nurses with extensive experience, as younger nurses might lack the requisite professional knowledge and skills. Particularly in terms of communication with parents and providing guidance, younger nurses often felt inadequate, which exacerbated the issue of insufficient human resources.

Nurses with limited experience cannot independently implement KMC. It requires nurses with a certain level of clinical experience and expertise, as the scope of communication and guidance involved is highly flexible. Younger nurses may only provide mechanical responses when communicating with parents, and they are unable to offer effective education or address queries. Handling unexpected situations is even more challenging for them. (H1)

#### 3.3.4 Inadequate parental compliance

The acceptance and compliance of parents were also mentioned as another barrier during the implementation of KMC. On the one hand, some healthcare providers [five participants (29%)] noted a lack of understanding coupled with misconceptions and cognitive dissonance regarding KMC among preterm infant parents. For instance, some parents did not grasp the necessity and benefits of KMC, mistakenly believing that hospitals offered KMC solely for profit-making purposes. These biases and misunderstandings brought psychological discomfort, leading to their resistance toward KMC.

Many parents may not fully understand the importance of KMC. Some mothers may even think it's just a way for the hospital to charge them; they think that such little infants know nothing. These cognitive biases make it difficult for them to persist with it. (H2)

On the other hand, healthcare providers [three participants (18%)] mentioned that some parents might discontinue their participation or inconsistently engage in it after the initial trial for various reasons, including feelings of fatigue, time conflicts, or the perception of no significant effects. These factors diminished the acceptance and involvement of parents in KMC, ultimately resulting in challenges in maintaining long-term commitment or deeming it unnecessary to continue.

Some parents come for the first time but don't want to return for the second time. They come today, but feel it's unnecessary to come tomorrow. In this way, the care plan is disrupted. (H7)

### 3.4 Theme 4: suggestions for KMC implementation

To promote and optimize the implementation of KMC, healthcare providers proposed a range of suggestions and improvement needs, hoping to comprehensively improve it through a multi-pronged approach. These included enhancing the equipment and environment, fostering collaboration and communication among healthcare providers, and acquiring support from hospital managers.

#### 3.4.1 Improving equipment and environment

Many healthcare providers [nine participants (53%)] highlighted that engaging in KMC was a hard and laborious task for parents, especially when faced with sub-optimal facilities or uncomfortable environments, which would directly influence their experiences. Therefore, it was recommended that the KMC implementation environment and equipment should be optimized, such as providing better privacy protection and adjustable reclining chairs, to create a more cozy, homely, and family-oriented KMC implementation space, thereby enhancing parental compliance and satisfaction.

The equipment for KMC needs to be improved anyway; the more comfortable, the better. Our KMC chairs should be both easy to disinfect and comfortable. (H15)

It's definitely better to have one private room per family and then optimize its environment. The layout should be warmer, more daily, and more family-friendly, and it would be great to play some music, creating a relaxed atmosphere. (H8)

#### 3.4.2 Strengthening collaboration between nurses and doctors

Apart from the above, some healthcare providers [six participants (35%)] highlighted the significant impact of the close collaboration between medical and nursing staff in KMC practice. Nurses observed that parents trusted doctors more and recommended that doctors should engage in KMC advocacy together with them to enhance parental compliance. Furthermore, cooperation among healthcare providers ensured the smooth delivery of information and coordinated KMC implementation plan, thereby improving the effectiveness and quality of KMC.

No matter how well we nurses do it, parents may not appreciate it. However, they all listen to doctors very attentively, so it's essential for doctors to be involved and convey this information to the families. (H14)

Doctors may need to participate in the KMC, either providing guidance or updating on the infants' condition. Some doctors may need a prompting reminder to be more proactive and involved. There is potential for further deepening their engagement in this aspect. (H9)

#### 3.4.3 Support from hospital managers

The majority of healthcare providers [11 participants (65%)] emphasized that support from hospital managers played a pivotal role in driving the practice of KMC. It was crucial for managers to acknowledge the significance of healthcare professionals in KMC and provide appropriate incentives for those involved in the practice. Among various incentive measures, performance bonuses were considered one of the most practical and effective ways, especially in the current shortage of human resources in NICUs. By integrating the KMC implementation into performance evaluations and providing bonuses, more nurses would be motivated to engage in KMC during their rest time, thereby alleviating the issue of manpower shortage to some extent.

If there are rewards, nurses will be more willing to do it, even on their rest days. So, the hospital should emphasize the labor value, and a performance bonus is the most realistic and tangible. (H5)

In addition to the material rewards, some healthcare providers [four participants (24%)] agreed that spiritual incentives were also crucial. These non-monetary incentives are mainly manifested in the praise and recognition of individuals involved in KMC to enhance their sense of honor and achievement. By publicly acknowledging and commending, managers could make healthcare professionals feel the importance and value of their contributions to KMC, thereby inspiring greater enthusiasm and dedication in their work.

The managers have praised our team's efforts at hospital weekly meetings several times. And they also extended invitations to staff from other medical facilities to visit us and learn about KMC practice. Such positive feedback serves as a strong source of encouragement for us. (H6)

Moreover, over half of the healthcare providers [nine participants (53%)] mentioned the vital importance of policy and institutional support from the hospital leadership level for KMC. They stated that if the medical and nursing departments jointly emphasized and actively promoted the implementation of KMC, it would undoubtedly be carried out effectively. This was because only with the top-down attention and comprehensive support from hospital managers could the hardware facilities and staffing keep up more smoothly, and the high execution ability of clinical nurses could be fully leveraged.

In Chinese hospitals, as long as the managers are willing to do and support it, it can definitely be implemented…When it comes to implementing KMC, from hospital managers to department managers to nurses, I think everyone should have the mindset to carry it out. (H17)

## 4 Discussion

Although healthcare providers generally acknowledged the significant benefits of KMC for preterm infants and their parents, there remained a lack of intrinsic motivation to implement KMC, mainly due to the lack of economic profitability. Moreover, other potential concerns arose in the process of KMC, such as worries about nursing risks, potential pressures and disputes stemming from parents' presence, and various obstacles to KMC implementation, such as inadequate human resources. These factors indicated that promoting the implementation of KMC in China faced significant challenges. Therefore, there was a pressing need to enhance collaboration among healthcare providers and gain support from hospital managers to facilitate the implementation of KMC in China.

This study showed that the lack of motivation among healthcare providers to implement KMC mainly stemmed from the economic unprofitability of departments and hospitals. In the market-oriented healthcare environment in China, hospitals need to be responsible for their own profits and losses, which means they are financially self-sufficient (47, 48). When deciding whether to implement a program or procedure, they needed to conduct a cost-benefit analysis. Healthcare providers perceived KMC charges as relatively low. Compared with the corresponding space, equipment, and manpower input, the expenses for KMC could not make ends meet. Moreover, healthcare providers' bonuses were directly tied to hospital income (49, 50), and the lack of economic incentives made them feel insufficiently motivated to implement KMC. This viewpoint represented the personal and subjective cognition of the healthcare providers we interviewed, reflecting their concerns and experiences related to the economic aspects of KMC implementation. Actually, some studies indicated that in environments with limited medical resources, KMC was recognized as the most straightforward and cost-effective method for managing preterm infants, especially when compared to the financial losses that may result from not providing KMC (51, 52). However, the extent to which KMC aligns with the long-term interests of hospitals in China, particularly in Zhejiang Province with relatively abundant medical resources (e.g., incubators) but high human resource costs, necessitates further research and validation. The core reason why hospital managers were reluctant to carry out KMC was not due to a lack of awareness of its clinical benefits but because KMC was not profitable in Chinese hospitals under the current pricing system. At present, with the gradual promotion of DRGs in major hospitals in China, hospitals might be more motivated and willing to provide nursing services such as KMC, which could shorten the average hospital stay (53). With the full roll-out of DRGs, hospitals would receive reasonable compensation for the costs associated with implementing KMC (54, 55), which is likely to be an opportunity for the future promotion of KMC.

Our study emphasized that insufficient human resources was a significant barrier that affected the implementation of KMC, which is similar to previous findings. A review on KMC in the sub-Saharan African region indicated that the shortage of healthcare workers limited the support available to parents, hindering the implementation of KMC (34). In countries such as Malawi and Indonesia, challenges related to human resources and concerns about increased workloads were reported as obstacles to implementing KMC (56, 57). The financial self-sufficiency attribute of Chinese hospitals meant that when allocating healthcare staff, hospitals needed to consider not only whether they could meet the medical workload but also the human efficiency, which was likely the underlying reason for the shortage of doctors and nurses in China. Compared to high-income countries like the United States, the ratio of nurses to preterm infants in China was relatively low (58, 59). However, a study indicated that while an initial increase in workload during the early stages of KMC implementation, continuous KMC implementation thereafter could reduce the nurse workload (56), which deserved further attention and exploration in future research. Additionally, if KMC were included in medical insurance, hospitals would be relieved of the burden of economic considerations when implementing KMC but rather focus on whether it was beneficial for preterm infants (60). Therefore, it is necessary to call on policymakers to include KMC in medical insurance, providing the necessary institutional guarantee and support for NICUs to carry out KMC.

In the absence of economic incentives, the key to successfully integrating KMC into routine practice laid in the support of hospital managers. This involved motivating healthcare staff, particularly nurses, to engage more effectively in KMC through enhancing their intrinsic drive and external support. Research showed that a lack of leadership's spiritual motivation or support could make healthcare workers more susceptible to wavering, which affects the implementation and participation in clinical operations (61). Their intrinsic motivation could be enhanced through hospital managers' recognition and reinforcement of their values (62). Therefore, managers should demonstrate the significance of KMC in the recovery of preterm infants and the wellbeing of parents to nurses, enabling them to perceive their contributions as valuable and appreciated. This internal cognition and motivation should have been considered a key part of healthcare personnel management and should have been taken into account when assessing staffing and implementation (27, 38). Studies on the introduction of KMC found that many nurses and doctors attributed their enthusiasm for KMC to the leadership and support of hospital managers (26, 60). Moreover, it was found in our study that the performance bonus was the most practical and effective support measure currently available, as evidenced in previous research (63). Therefore, incorporating the KMC implementation into performance appraisal could enhance the enthusiasm and involvement of healthcare workers in KMC.

Our findings indicated that there were differences in the perceptions of KMC between healthcare providers and parents of preterm infants. According to the literature, parents' cognition and evaluation of KMC directly influenced their decisions and behaviors, including their willingness to accept KMC and their degree of involvement in the implementation process (31). Although healthcare providers believed that KMC charges were relatively low and even resulted in department losses, it was purely for the benefit of preterm infants and parents; on the other hand, many parents failed to realize the significance and benefits of KMC and misunderstood medical staff implementing KMC to make a profit. To some extent, this was also related to the fact that KMC was not included in the medical insurance; non-insured medical procedures were easy to be regarded as unnecessary by parents and provided by hospitals solely for profit-driven purposes (64). The cognitive gap in KMC between healthcare providers and parents of preterm infants stemmed from information asymmetry between the two parties. Breaking down these cognitive barriers to achieve better understanding was crucial (32). This also highlighted the inadequacy of healthcare providers' early KMC education efforts. A study suggested to introduce the concept of KMC during prenatal care or immediately after delivery (65). Meanwhile, healthcare providers should enhance KMC advocacy and education to promote long-term parental engagement and compliance.

Our study also found that some healthcare providers harbored negative emotional experiences of KMC, believing that the presence of parents would increase the risk of nursing safety and medical disputes. This negative attitude hindered staff from actively engaging in KMC. However, previous research showed that parental involvement in KMC could promote the recovery of preterm infants, enhance parental understanding of preterm infant-related knowledge, and teach them some care-giving skills (66, 67), while KMC did not increase the incidence of infants' infections in NICUs (68). In our study, some healthcare providers perceived parental involvement in KMC as a burden, which was obviously one-sided and biased. This perception might be related to the fact that Chinese NICUs traditionally operated as unaccompanied wards, with staff having limited contact with parents and being unaccustomed to working in front of parents (69). To some extent, healthcare providers' concerns about the potential risks of medical disputes when operating under parental supervision or monitoring confirmed the tense doctor-patient relationship in China (70). While excluding parents may have mitigated certain risks for healthcare providers, it also meant missing an opportunity to establish a trusting relationship with parents, which could have posed obstacles to specific tasks later on (71). Therefore, it is of great significance to shift the mindset of healthcare providers, enhance communication and trust with parents, and alleviate concerns about the presence of parents.

On the other hand, some healthcare providers in our study expressed that they could sense the increasing satisfaction from parents and the gradual improvement in cooperation with each other, which in turn strengthened their intrinsic motivation to carry out KMC. In other words, some medical staff had also become aware of the positive aspects from parents of preterm infants. It is indeed natural for healthcare providers to hold conflicting feelings or attitudes about the parent's presence during KMC, given their differing perspectives and experiences. Therefore, in promoting KMC, healthcare providers should not solely concentrate on the potential risks associated with parental involvement, but rather raise awareness of the positive aspects of parental involvement. Studies showed that healthcare providers' knowledge, beliefs, and attitudes toward KMC were crucial for its implementation (37, 72). When staff in NICUs feel that they are assisting preterm infants and their parents in resolving issues through KMC, they would not perceive parental presence as a nuisance or a disadvantage. Conversely, if healthcare providers are merely going through the motions of their tasks, they may view the presence of parents as disruptive and inconvenient. Studies involving healthcare providers and parents participating in KMC demonstrated that as parents became more familiar with daily nursing, both parties experienced reduced anxiety, leading to an improvement in the doctor-patient relationship and a closer bond (73, 74). As mentioned by the interviewees in our study, they sensed the trust and recognition from parents being elevated, the cooperation between staff and patients gradually being improved, and a solid emotional connection was established between them. Therefore, healthcare providers need to recognize the positive significance and value behind parental involvement, viewing it as an essential way to promote the recovery of preterm infants and enhance relationships with parents. This positive feedback will further enhance their motivation to implement KMC.

Understanding the barriers and challenges to implementing KMC in specific settings is the foundation for successful implementation. In our study, some participants pointed out that the current training related to KMC was insufficient, and the standardization still needed further improvement and uniformity. Considering that the personnel implementing KMC in China were relatively not fixed, the unified operations could not only make the entire KMC process more standardized, systematic, and comprehensive but also avoid the waste of manpower and resources (26, 64). Therefore, it is necessary to localize the existing KMC guidelines based on evidence-based methods and establish standardized practices for KMC implementation that are suitable for the Chinese context. Furthermore, through systematic training and policy support, healthcare providers can acquire the necessary knowledge and skills, creating a more conducive environment for KMC implementation, reducing their concerns, and boosting the team's confidence in KMC practice (75). Existing studies indicated that these standards and training programs should at least cover aspects such as how to properly implement KMC, how to deal with potential risks, and how to communicate with parents effectively (76–78). In this way, tailored support strategies should be developed from the perspective of healthcare providers to promote the implementation of KMC and expand the scale of KMC practice in China.

### 4.1 Limitations

This study used a qualitative research method to explore the perceptions and experiences of Chinese healthcare providers in caring for parents' KMC of preterm infants in four NICUs in China. However, the study had several limitations. First, although the researchers and participants were in a quiet and undisturbed office during the interview, it was inevitable that participants would be interrupted by calls they needed to answer or other tasks requiring urgent handling, which may have impacted their thinking during the interview. Second, all hospitals included in the study are tertiary hospitals, including general teaching hospitals and maternal and child healthcare hospitals located in major urban cities in Zhejiang. The experience and practice of KMC in lower-level healthcare facilities may be different.

## 5 Conclusions

Our study showed that although healthcare providers generally agreed on the importance and benefits of KMC for infants and parents, after considering multiple factors, they believed that implementing KMC was not cost-effective and lacked intrinsic motivation for implementation. These factors included the fact that KMC was not profitable at the economic level, concerns about potential risks due to the presence and participation of parents, and obstacles such as insufficient human resources and lack of norms in the implementation. It is recommended that hospital managers provide policy support in performance appraisal and recognize and encourage healthcare professionals so as to enhance their motivation to implement KMC. Promoting parental compliance with KMC also requires collaboration between medical and nursing staff. Moreover, it is also necessary to improve the training of staff and standardize the KMC program so as to form a more conducive and supportive KMC implementation environment.

## Data availability statement

The original contributions presented in the study are included in the article/Supplementary material, further inquiries can be directed to the corresponding author.

## Ethics statement

The studies involving humans were approved by Ethics Committee of the Women's Hospital, School of Medicine, Zhejiang University. The studies were conducted in accordance with the local legislation and institutional requirements. Written informed consent for participation in this study was provided by the participants' legal guardians/next of kin.

## Author contributions

QC: Writing – original draft. YZ: Writing – review & editing. MH: Writing – review & editing. DC: Writing – review & editing. XX: Writing – review & editing.
