# Supplementary material for: Healthcare providers' perceptions and experiences of kangaroo mother care for preterm infants in four neonatal intensive care units in China: a qualitative descriptive study
Source: Front Public Health. 2024 Jul 8;12:1419828. doi: 10.3389/fpubh.2024.1419828 (PMC11260804; doi:10.3389/fpubh.2024.1419828)
Supplement: Supplementary file 1 [file Table_1.DOCX]

Supplementary Material

# Supplementary Tables

**Supplementary Table 1** Semi-structured interview guide for doctors and nurses

| **Interview questions to doctors and nurses in NICU** |
| --- |
| 1. What are the advantages and disadvantages of parental involvement in KMC for preterm infants? What is your understanding of KMC for preterm infants? |
| 2. Please describe how you manage parental involvement in KMC for preterm infants in your daily work？How effective do you think this management is? |
| 3. How do you feel in the process of guiding parents of preterm infants to participate in KMC? Please share a specific case. |
| 4. What confusions or difficulties have you encountered in the process of guiding parents of preterm infants to participate in KMC? How did you resolve the difficulties you encountered? |
| 5. How did you learn about knowledge of KMC for preterm infants? How do you think about the training provided in your department on KMC? How did this affect your work? |
| 6. What problems do you think still exist in the implementation of KMC for preterm infants? Do you have any suggestions for improving the implementation of KMC? |
| 7. What additional information or suggestions can you provide to better guide parents in participating in KMC for preterm infants in the future? |

Abbreviations: KMC, kangaroo mother care; NICU, neonatal intensive care unit

**Supplementary Table 2** Semi-structured interview guide for head nurses

| **Interview questions to head nurses in NICU** |
| --- |
| 1. How do you manage nursing staff involved in the participation of KMC for preterm infants? How do you feel about the current implementation of KMC and the management of nursing staff? |
| 2. What are the advantages and disadvantages of parental involvement in KMC for preterm infants? What potential risks may be involved? How do medical staff generally respond? |
| 3.From your perspective as a manager, what are the main problems that healthcare professionals currently face in guiding parents of preterm infants in practicing KMC? What are your recommendations to address these problems? |
| 4. What are the current challenges faced by your department in implementing KMC for preterm infants? What are the facilitators and barriers to KMC implementation (from perspectives of parents, healthcare providers, and the hospital, etc.)? |
| 5. Have the medical staff in your department received training on KMC for preterm infants? Where do the guidelines for KMC training originate? What are the current shortcomings of training on KMC and how can it be improved? |
| 6. Do you have any additional suggestions to better equip nurses in the future for guiding parents in KMC for premature infants? |

Abbreviations: KMC, kangaroo mother care;NICU, neonatal intensive care unit
